# The Occurrence of Skin Mites from the Demodecidae and Psorergatidae (Acariformes: Prostigmata) Families in Bats, with a Description of a New Species and New Records †

**DOI:** 10.3390/ani12070875

**Published:** 2022-03-30

**Authors:** Karolina Cierocka, Joanna N. Izdebska, Leszek Rolbiecki, Mateusz Ciechanowski

**Affiliations:** 1 Department of Invertebrate Zoology and Parasitology, Faculty of Biology, University of Gdańsk, Wita Stwosza 59, 80-308 Gdańsk, Poland; karolina.cierocka@ug.edu.pl (K.C.); leszek.rolbiecki@ug.edu.pl (L.R.); 2 Department of Vertebrate Ecology and Zoology, Faculty of Biology, University of Gdańsk, Wita Stwosza 59, 80-308 Gdańsk, Poland; mateusz.ciechanowski@ug.edu.pl

**Keywords:** checklist, Chiroptera, *Demodex pusillus* sp. nov., skin mites, *Nyctalus noctula*, parasites, *Plecotus auritus*, *Psorergatoides kerivoluae*, topography

## Abstract

**Simple Summary:**

This paper describes a new species, *Demodex pusillus*, inhabiting the hairy skin of *Nyctalus noctula*, which is one of the smallest arthropods. New data on the coexistence of skin mites from the sister families Demodecidae and Psorergatidae in bats are also included, as well as an updated global checklist and data on their occurrence, including location (topography) within the hosts.

**Abstract:**

The bat skin mites from the closely-related Demodecidae and Psorergatidae families occur synhospitally, populating the same host species and perhaps neighboring microhabitats. However, data on their occurrence and parasitism are fragmentary and dispersed. Thus far, 27 Demodecidae and 18 Psorergatidae species have been described, but the coexistence of mites from both families was only demonstrated in six species of bats. This article presents a description of *Demodex pusillus* sp. nov. from *Nyctalus noctula*, including a new host record (first observation of demodecid mites in *Nyctalus*) and a new record concerning the occurrence of *Psorergatoides kerivoluae* in *Plecotus auritus*. It also includes an updated global checklist of the occurrence of Demodecidae and Psorergatidae in Chiroptera, including data on their records/distribution and location in their hosts. In both studied families, the mites exhibit preferences, and even topographic specificity, colonizing different microhabitats in the host, including the eye region (e.g., Meibomian glands of the eyes, corneal surface and eyelid vault), wing membranes and hairy skin on the body. Such colonization of separate microhabitats enables different species to co-occur within the same host, while the total number of parasites determines the level of parasite load, with higher levels being associated with the incidence of disease symptoms. It is worth mentioning that *Demodex pusillus* sp. nov. is the smallest known representative of the Demodecidae family and one of the smallest animals (70–80 micrometers in length).

## 1. Introduction

Over 20 mite families are associated with bats. Within this group is a separate ecological group comprising the skin mites, which are stationary parasites whose entire life cycle takes place within the host body [1,2]. This subgroup includes the closely-related sister families Demodecidae and Psorergatidae, whose members are mostly characterized by high host specificity, being monoxenic or oligoxenic parasites [3,4,5]. It is probable that representatives of these families can occur synhospitally, i.e., colonizing different habitats in the same hosts. However, data on their occurrence and parasitism are fragmentary and dispersed. Thus far, they have been observed in relatively few bats from different parts of the world, and only six bat species have been found to have representatives of both mite families [4,5]. However, these relationships have never been analyzed at individual level and it remains unknown whether mites from either family can occur concomitantly, or if they compete for the same, or similar, microhabitats, and the presence of one group excludes colonization by representatives of the other. 

No such competition has been observed for the Demodecidae, where individual species inhabit distinct microhabitats within the host at the same time. Six species of bat have been found to harbor one or more Demodecidae species [5], and such co-occurrence has also been noted at the individual level, e.g., individual bats were found to demonstrate both *Demodex chiropteralis* (hairy skin of the body, head) and *D. plecoti* (wing membranes) [6]. Similar examples of co-occurrence have also been observed for *D. mystacina* and *D. novazelandica*, inhabiting adjacent microhabitats within the eyelids of *Mystacina tuberculata*, and for *D. neopisthosomae* and *D. spelaea*, which both inhabit the Meibomian glands of *Eonycteris spelaea* eyelids [7,8].

The lack of data on the co-occurrence of Demodecidae and Psorergatidae is related to the methodological difficulties in their research, more precisely, their minuscule size, identification issues and secretive lifestyle. While these mites are more easily detected when their presence elicits disease symptoms, this is a rare phenomenon, and they typically occur asymptomatically [9]. Analyses of asymptomatic cases are highly labor-intensive and they only include select locations or part of the body surface (cuttings/skin fragments), low number of hosts (difficult to obtain) and typically mites from one group/family. 

The present study analyzed the occurrence of skin mites from both families in the common noctule *Nyctalus noctula*, as well as brown long-eared bat *Plecotus auritus*. All studied bats had previously been confirmed to demonstrate the asymptomatic presence of Demodecidae or Psorergatidae. The study presents new data on the occurrence of these mites, including the discovery of a new species described as *Demodex pusillus* sp. nov.

## 2. Material and Methods

### 2.1. Detection of Skin Mites in Bats

Six specimens of dead *Nyctalus noctula* (Poland, Pomerania, Redzikowo near Słupsk, 54°28′21.48″ N/17°06′13.27″ E) collected from November 2007 and six specimens of dead *Plecotus auritus* (Poland, Pomeranian Voivodeship, Gdańsk, 54°25′32″ N/18°29′29″ E, 1 bat; Gdynia, 54°31′57″ N/18°27′11″ E, 54°29′01″ N/18°32′27″ E, 2 bats; Skrzeszewo, 54°17′53.38″ N/18°20′24.02″ E, 2 bats; Zbysław, 54°14′43.79″ N/17°28′31.64″ E, 1 bat), collected from February 2012–August 2018, were examined for Demodecidae and Psorergatidae mites. All *N. noctula* drowned during rain after falling into the gutter on residential block and were found a day later, while *P. auritus* were found during routine winter bat census in the underground roosts and thus, probably died during hibernation.

The skin mites were isolated using skin digestion methods [10], with modifications to suit the examined host. For analyzing the topography (microhabitats) of mites, skin fragments of 1 cm^2^ were examined from several body regions, including the head (around the eyes, ear pinnae, nose, lips, chin, cheeks, vertex), neck, abdomen, back, wing membranes, limbs and genital–anal area. Skin samples were preserved in 70% ethanol and digested in 10% KOH solution. The obtained samples were decanted (the examination of 1 cm^2^ of skin was equal to that of approximately 100 wet preparations) and examined using phase-contrast microscopy (Nikon Eclipse 50i, Nikon Corporation, Tokyo, Japan). Mites were placed in polyvinyl–lactophenol solution and subjected to morphometric examination. All measurements are in micrometers and were obtained as follows: total body length = length of gnathosoma, podosoma and opisthosoma; gnathosomal width = width at base; podosomal and opisthosomal width = maximum width. 

Specimen depositories are cited using the abbreviation UGDIZP, University of Gdańsk, Department of Invertebrate Zoology and Parasitology, Gdańsk, Poland [11]. 

The description of the species adopted the nomenclature commonly used for the family Demodecidae [12] and was completed with the nomenclature proposed by Bochkov [3] for the superfamily Cheyletoidea, and by Izdebska and Rolbiecki [13]. 

The prevalences were calculated to determine the level of host infection [14]. 

### 2.2. The Checklist Structure

The checklist was drawn up based on manuscripts published during the period 1859–2019. It also contains own unpublished data, marked in the table as the present study. Demodecidae and Psorergatidae species have been arranged in systematic order, and in alphabetical order within the genera. The list includes all formally described species and information on dates of host species, as well as the occurrence have been included. 

The scientific and common names of the hosts follow Wilson and Reeder [15] and the Integrated Taxonomic Information System [16].

## 3. Results

### 3.1. Descriptions

#### *Demodex pusillus* Izdebska, Cierocka, Rolbiecki et Ciechanowski, 2022

**Figure 1:**
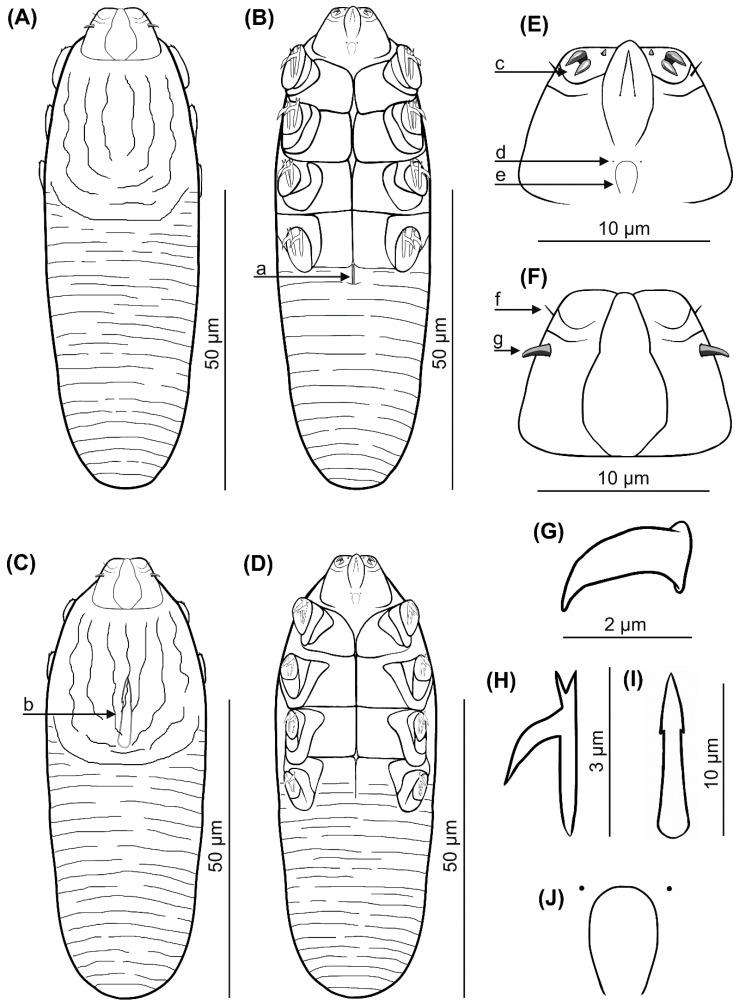
*Demodex pusillus* sp. nov.: female, dorsal view (**A**), female, ventral view (**B**), male, dorsal view (**C**), male, ventral view (**D**), gnathosoma, female, ventral view (**E**), gnathosoma, female, dorsal view (**F**), supracoxal spine, lateral view (**G**), claw on the leg (**H**), aedeagus (**I**); pharyngeal bulbs with subgnathosomal setae (**J**); a: vulva, b: aedeagus, c: spines on palps, d: subgnathosomal seta (seta *n*), e: pharyngeal bulb, f: seta *v”F*, g: supracoxal spine (seta *elc.p*).

**Figure 2:**
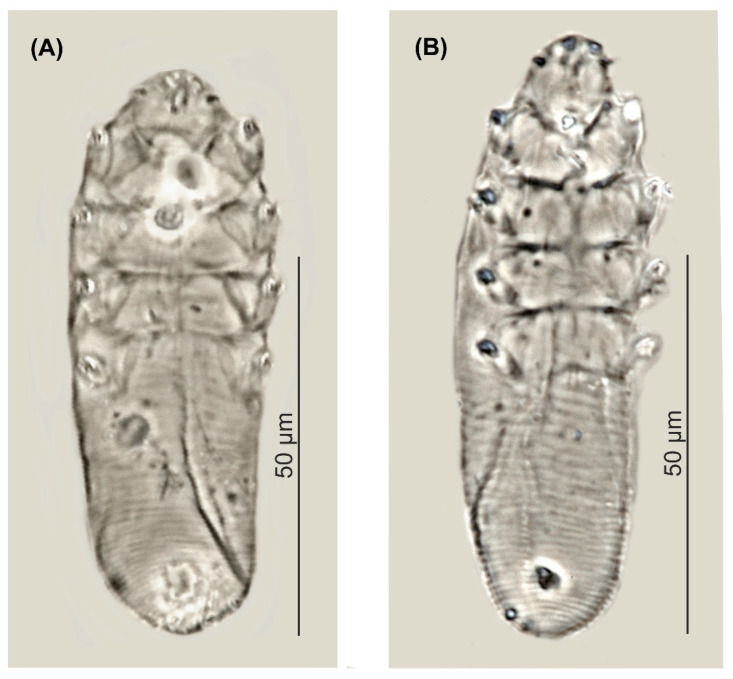
*Demodex pusillus* sp. nov.: male (**A**) and female, holotype (**B**).

Female (*n* = 1 holotype and 27 paratypes): The female body is stocky, cylindrical with short gnathosoma and podosoma similar in length and width to opisthosoma, 80 (70–93) long and 25 (23–30) wide (holotype, 78 × 24) (Table 1, Figure 1 and Figure 2). Gnathosoma trapezoidal are shorter than the base width. On the dorsal side at the external edge, a pair of hook-shaped supracoxal spines (setae *elc.p*) present, ca. 2.0 long (holotype, 2.0) and are directed outwardly. Palps 3-segmented terminate in three spines (two larger, curved and one small, conical) on the tibio–tarsus; also, setae *v”F* on the middle segment (trochanter–femur–genu) present. On the ventral part of gnathosoma, horseshoe-shaped pharyngeal bulb, with a pair of small subgnathosomal setae (setae *n*) are situated anterior on both sides. The podosoma rectangular. Four pairs of short legs, with coxa integrated into the ventral idiosomal wall and five free, overlapping segments (trochanter-tarsus); two forked claws, ca. 3.0 long (holotype, 3.0), with large, hooked spur on each tarsus. Epimeral plates (coxal fields) are trapezoidal and sclerotized; all epimeral plates connect medially; posterior edges of pair IV form a triangular incision. On the dorsal side of the podosoma, a podosomal shield is present, reaching the anterior level of legs III. The opisthosoma oval constitutes 45 (41–49) of body length (holotype, 45). Whole opisthosoma is distinctly annulated; annuli relatively wide ca. 1.0–1.5. The opisthosomal organ is absent. The vulva 4 (3–5) long (holotype, 3.0) is located in incision of IV epimeral plate.

Male (11 paratypes): On average, males are slightly smaller than the females, 75 (68–83) long and 24 (23–28) wide. Gnathosoma trapezoidal are shorter than the base width. Pharyngeal bulb and morphological details of gnathosoma are similar to those in females. The shape of podosoma and legs are similar to those in the females, but the posterior edge of epimeral plate IV is without a triangular incision. A podosomal shield is present, also reaching the anterior level of legs III. The opisthosoma constitutes 47 (43–56) of body length, is oval, clearly annulated and with relatively wide annuli, ca. 1.0–1.5. The opisthosomal organ is absent. The aedeagus 11 (9–13) long, on the dorsal side, located between epimeral plates II and III. The genital opening is located on the dorsal surface, at the level of anterior edge of the epimeral plate II.

Type of material: The female holotype (reg. no. UGDIZPVNnDDp01f) was from *Nyctalus noctula* (reg. no. MCVNn01/2007-06/2007), Redzikowo near Słupsk, Pomeranian Voivodeship, Poland, November 2007, parasites coll. K. Cierocka, J.N. Izdebska, L. Rolbiecki; host coll. M. Ciechanowski; 27 female paratypes (reg. nos. UGDIZPVNnDDp02–28f) and 11 male paratypes (reg. nos. UGDIZPVNnDDp01–11m) were from *Nyctalus noctula* (reg. nos. MCVNn01/2007-06/2007), Redzikowo near Słupsk, Pomeranian Voivodeship, Poland, November 2007; the collectors are the same.

Type of material deposition: The whole-type material (mounted microscope slides with the demodecid mites) was deposited in the scientific collections within the framework of the Collection of Extant Invertebrates in Department of Invertebrate Zoology and Parasitology, University of Gdańsk, Poland (UGDIZP).

Infection and location in the host: *Demodex pusillus* sp. nov. was found in all examined common noctule (100%); 39 specimens in total were found (11 males, 28 females). The demodecid mites were found on the hairy skin of the body (head—6 individuals, abdomen—6, back—27). The observed mites did not cause any lesions in examined common noctule.

Etymology: The specific epithet *pusillus* refers to the small size of this demodecid mite.

### 3.2. Differential Diagnosis

Among the known Demodecidae, *D. pusillus* sp. nov. is close to *D. plecoti*, and described from another European representative of the Vespertilionidae, namely the brown long-eared bat (Table 1 and Table 2). However, *D. pusillus* sp. nov. is smaller, has different body proportions (*D. pusillus* sp. nov. is cylindrical, while *D. plecoti* – fusiform) and does not exhibit sexual dysmorphism typical of *D. plecoti*; in *D. pusillus* sp. nov., males are typically slightly smaller than females; in *D. plecoti*, males are clearly smaller than females and their epimeral plates are shaped and arranged differently to females. The gnathosoma of *D. pusillus* sp. nov. is trapezoid shaped and oval in *D. plecoti*. Supracoxal spines in both species are conical, hook-shaped/curved, but in *D. pusillus* sp. nov. they are located in the anterior part of the coxal (basal) palp segment, near the edge and directed outwards, while in *D. plecoti*, they are located in the middle of the coxal palp segment and directed downwards, to the inside (posteromedially). The terminal segment of the palpi has three spines in *D. pusillus* sp. nov. (two larger, curved, one small, conical), and two large spines in *D. plecoti* (one bifurcated and other simple, conical). Subgnathosomal setae in *D. pusillus* sp. nov. are situated at the level of the anterior edge of the pharyngeal bulb, but are situated lower in *D. plecoti*. In both sexes, all epimeral plates connect medially in *D. pusillus* sp. nov., but are separated in *D. plecoti* (pairs I and IV of epimeral plates partly come into contact in females, but only pair I in males). In *D. pusillus* sp. nov., the tarsi of the legs are equipped with forked claws, with a long spur, while in *D. plecoti*, the claws are also forked, but lack the spur. The aedeagus of the male *D. pusillus* sp. nov. is situated at the level of the pair II–III epimeral plates (genital orifice at the level of anterior edge of II epimeral plate), while it is located between plates III and IV in *D. plecoti* (genital orifice—at the level of border between plates II and III). The vulva is located in an incision between pair IV epimeral plates in the female *D. pusillus* sp. nov., but below the posterior edge of pair IV in *D. plecoti*. The typical microhabitat is also different: *D. pusillus* sp. nov. was found in the hairy skin of the body, and *D. plecoti* in the wing membranes.

### 3.3. A New Record of Psorergatidae

**Figure 3:**
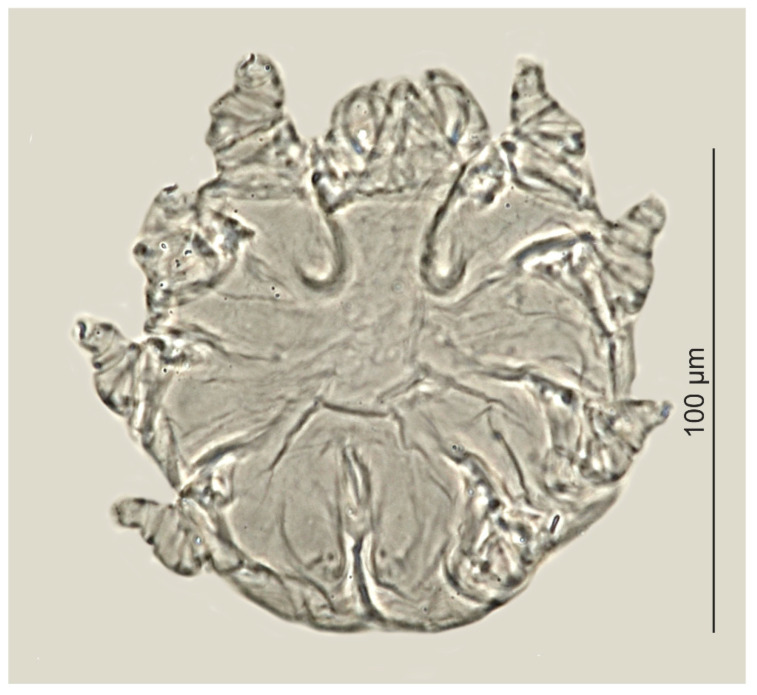
*Psorergatoides kerivoluae*, female.

*Psorergatoides kerivoluae* was found in one out of the six examined brown long-eared bats (Table 3, Figure 3). Overall prevalence was 16.7%, with two individuals of *P. kerivoluae* (females) being found in the forehead region and in the ear canal. No skin lesions were observed in the infested bat.

The voucher specimens were deposited in the scientific collections within the UGDIZP scientific collection.

### 3.4. Biodiversity of Demodecidae and Psorergatidae in Chiroptera 

In the 55 studied bat species from 11 families, 45 skin mites from Prostigmata were found, including 28 Demodecidae and 18 Psorergatidae. The highest number (12 species) was found in bats classified from the Vespertilionidae family (Table 4).

### 3.5. Co-Occurrence of Demodecidae and Psorergatidae

All examined bats were found to have skin mites. Among *N. noctula*, six individuals were infested with *D. pusillus* sp. nov.; *Psorergatoides nyctali* had previously been recorded in two of these individuals (retrospective study, [43]). The infestation level was low (only single individuals were found); no skin lesions caused by the presence of mites could be observed. In turn, out of the six *P. auritus* examined in the present study, one was found to have *P. kerivoluae*. Earlier, the same bat individual was found to harbor *D. chiropteralis* [23] and *D. plecoti* [6]. 

Mites from both families have been found in seven bat species. In addition, six bat species featured at least two Demodecidae species, with the highest number found in *Carollia perspicillata*: four species from three genera. Only one or two Psorergatidae species were observed. Mites from individual species exhibited clear topographic and topical preferences, with a high diversity of microhabitats: the parasites inhabited the head region (eyelids, including Meibomian glands, eye, including the corneal surface, eyelid vault and hairy skin of the head), hairy areas of the body, wing membranes and non-hairy (membranous) skin regions (Table 4).

## 4. Discussion 

Little is known on the co-occurrence of related and ecologically-similar skin mite families from the Demodecidae and Psorergatidae in the same host, as evidenced by the lack of studies in the global literature. Analysis of host records (Table 4) indicates that these mites demonstrated synhospital occurrence in seven chiropteran species, with representatives of both families being present in each individual. In the present study, these findings are supplemented with findings in *Nyctalus noctula*, which were found to harbor both the previously known *Psorergatoides nyctali*, and a new species, *D. pusillus* sp. nov. 

In addition, individuals of *D. chiropteralis* were found next to *D. plecoti* and *P. kerivoluae* in *Plecotus auritus*, confirming that mites from both families can co-occur in the same host. These mites occupied both distant and adjacent microhabitats within their hosts, exhibiting low density in the skin (low infestation intensity). Thus, balanced host–parasite relationships developed, without burdening the host, not causing disease symptoms and thus not manifesting their presence. These mites could hence only be detected by means of a labor-intensive digestion and decanting method, consisting of searching subsequent fragments of the entire skin surface.

Occurrence of host specific (monoxenic) parasitic mites, inhabiting different microhabitats within their hosts, comes as a rule for Demodecidae [5]. Although they most likely demonstrate a common occurrence within host populations, and their geographic distribution corresponds to the distribution of host species, their difficult detection results in their presence being sporadically recorded and described, particularly in wild, rare and protected animals [46]. The majority of demodecid mites species are known solely from individual records [47]. For example, *D. chiropteralis*, first described from the United Kingdom, was only found for the second time after one hundred years in Poland. In addition, despite a number of studies, only one species from the Psorergatidae, *P. nyctali*, has been found in *N. noctula*, known from only two records [42,43]. The present study brings new data on the occurrence of a Demodecidae representative in this bat species, which constitutes a new host record for the genus *Nyctalus*. The individuals found differ from the known Demodecidae and are described as a new species, *D. pusillus* sp. nov. The mite is associated with various regions of the hairy skin of the body; as such, it is likely to be the predominant species of this group in the common noctule. 

The Demodecidae populate different microhabitats within their hosts, the distance/extensiveness of which determines the possibility for reproduction and spread of the mites. In many mammal species, one Demodecidae species is usually found in greater numbers than others, inhabiting more limited microhabitats. For instance, in the house mouse *Mus musculus*, seven specific Demodecidae taxa are known, with the most common and numerous being *D. musculi*, inhabiting the hairy skin of the body, whereas other demodecid mite species are restricted to narrow microhabitats (e.g., vibrissae follicles, ear canals, tongue) and are rarer and less numerous [48]. It is likely that the demodecid mite described in the common noctule in the present study may be the predominant species from this group; however, it does not complete the list of potential future discoveries. 

An interesting observation was the record of *P. kerivoluae* in *P. auritus,* which was previously described on the basis of individuals obtained from *Kerivoula cuprosa* and *K. lanosa* from Congo [17]. Subsequently, *P. kerivoluae* was recorded from *P. auritus* in Belgium and Poland. Moreover, it has been recorded in five other vespertilionid bat species: *Myotis muricola* (Borneo)*, M. bocagii* (Republic of Côte d’Ivoire)*, M. myotis* (Poland)*, M. mystacinus* (Malaysia–questionable host) and *M. macropus* (Australia) (Table 4). The Psorergatidae are characterized by high host specificity, i.e., they are mono- or oligoxenic. One parasite species is usually noted in typically one host species or in several, closely related hosts (typically of the same genus) [4,49]. Therefore, *P. kerivoluae*, which thus far has been recorded in bats from three genera (although belonging solely to one family, Vespertilionidae), has a unique, wider range of host specificity compared to the rest of the Psorergatidae. This parasite has been found within the wing membranes, where it sometimes causes skin lesions in the form of several millimeters of white dots, scabs and convex, desquamating cysts, which facilitate its detection [39,40,41]. In such cases, only few individuals have typically been found; however, because they were only obtained in these studies from superficial scrapings, often collected from live individuals, the actual infestation state is difficult to ascertain. The wing membranes [17,36,37,39,42] are also the most commonly recorded location for other *Psorergatoides*, but these parasites have also been recorded in the pinnaes, on the outer side of ears, in the nasal membrane, on tail and limbs [17,36,39]. An astonishingly vast geographical range of that mite (covering Palaearctic, Afrotropic, Indomalayan and Australasian regions) and partially non-overlapping geographical ranges of the particular host species suggest that *P. kerivoluae* may, in fact, consist of several taxa, and needs revision.

The vast majority of these observations are related to the occurrence of skin changes. Similar observations have been made for most of the described Demodecidae taxa, whose presence is known to cause nodules, cysts, eyelid swelling or blepharitis, and which have enabled detection of these mites [19,26,28]. However, it should be kept in mind that through evolution, skin mites have adapted to functioning in hosts by creating stable host–parasite relationships with the lightest possible effect on host functioning. As such, parasitoses (*demodecosis, psorergatosis*) are very rare, and their development is typically determined by reduced immunity or the poor condition of their hosts [5]. Therefore, detection and discovery of these parasite species, their biology and aspects of their parasitism is of a random nature, often based on singular observations. 

Bats constitute the second most species-rich order within mammals (after rodents) [50,51], and their characteristic capability for active flight enables a relatively easy spread of their geographic distribution. Their particular species specializes in the utilization of different food (insects, vertebrates, blood, fruits, nectar and pollen), roosts (caves, trees, buildings and other anthropogenic structures) and strategies for survival during harsh seasons (hibernation and seasonal migrations). Even in our material, the two studied species, although both are insectivorous, represent different ecological adaptations. *Nyctalus noctula* is an open-space aerial hawker and long-distance seasonal migrant, hibernating mostly in hollow trees and parts of buildings above the ground, while *P. auritus* is a close-space foliage gleaner and sedentary species, hibernating mostly in underground roosts (caves, fortifications and cellars) [52]. It is hence only to be expected that the evolutionary success, ecological diversity and complicated body topography (membranes, ears, tragi and nose-leaves) of this group should be reflected in their equally high diversity of skin parasites, particularly when they occur asymptomatically and do not cause a burden for the host, not exceeding its tolerance threshold in terms of numbers. However, bats constitute an ecologically-separated group, compared to other mammals. The parasite transfer may occur on a significant scale solely within a given roost (between different, co-occurring bat species or genera) or within populations (between individuals of the same species), although bats may switch roosts regularly and change social behavior during their seasonal life cycle (spending time with different individuals during pregnancy and lactation, mating and hibernation). Bats are often present at high population densities within relatively small spaces due to their common roosting and tendency to form large groups in summer (nursery colonies), autumn (mating groups) and winter, even if their population densities in larger, landscape scales are unusually small for such small mammals. The development of social, even altruistic behavior in bats, would better enable skin parasites to colonize new hosts and, for some groups of mites, to become more specialized, especially those associated with bats for a longer period of time [53]. 

Such skin parasites include several genera found only in bats, such as *Pterodex*, and *Psorergatoides* and those known mainly from these mammals (*Ophthalmodex, Stomatodex*) [4,5]. The systematic diversity of skin mites appears to be greater among bats than for other mammals; however, this is not reflected by the number of species described, and this is undoubtedly a result of the generally poorer research status of skin mites in these hosts. Interestingly, the majority of the data come from Africa, South America and Asia, where local research on bats has typically addressed the acarofauna. In contrast, only a handful of studies have been devoted to the occurrence of these parasites in bats from Europe (Table 4). Despite the high interest in chiropterology, only six studies published in the 21st century have contained original data on skin mites in bats [6,23,25,41,42,43]. The explanation of that pattern may lie in the conservation status of Chiroptera that are not only legally protected but considered charismatic taxa, thus the majority of recent studies do not include deliberate collection of any specimen. Most material of arthropods parasiting on bats is, therefore, restricted to taxa collected from the body surface of living, captured-and-released individuals (Diptera: Nycteribiidae, Streblidae; Siphonaptera, Heteroptera, Acari: Spinturnicidae, Macronyssidae, Trombiculidae), while those living inside the integument (Demodecidae and Psorergatidae) are collected almost exclusively from randomly found dead individuals.

## 5. Conclusions

Considering the state of research on the occurrence of skin mites from Demodecidae and Psorergatidae families in other mammal orders, it is highly likely that the true number of these parasites in bats is much greater, and that their host circle among Chiroptera is more extensive. Only the recognition of the species diversity of these mites in bats will allow for a more complete analysis of the parasite–host systems and clarification of the issue of coexistence. 

## Figures and Tables

**Table 1:** Body size (mean, range and SD, in μm) for adults of *Demodex pusillus* sp. nov.

Morphologic Features	Males (*n* = 11)	Females (*n* = 28)
Length of gnathosoma	10 (9–10), SD 0.5	10 (8–12), SD 1
Width of gnathosoma (at base)	12 (12–14), SD 1	13 (12–14), SD 0.4
Length of podosoma	31 (27–33), SD 2	34 (28–38), SD 2
Width of podosoma	24 (23–28), SD 2	25 (23–30), SD 1
Length of opisthosoma	34 (32–40), SD 2	36 (33–45), SD 3
Width of opisthosoma	24 (22–25), SD 1	24 (23–28), SD 1
Aedeagus	11 (9–13), SD 1	-
Vulva	-	4 (3–5), SD 1
Total length of body	75 (68–83), SD 4	80 (70–93), SD 5

SD: standard deviation.

**Table 2:** Morphometric comparison between *Demodex pusillus* sp. nov. and *Demodex plecoti*.

Feature/Species	*Demodex pusillus* sp. nov.		*Demodex plecoti*	
Source	Present Study		Izdebska et al. [6]	
Sex	Males	Females	Males	Females
Sample size	(*n* = 11)	(*n* = 28)	(*n* = 40)	(*n* = 61)
Body total length	75 (68–83), SD 4	80 (70–93), SD 5	96 (80–109), SD 6	132 (118–158), SD 9
Body total width	24 (23–28), SD 2	25 (23–30), SD 1	35 (28–42), SD 3	41 (34–48), SD 4
Body length to width ratio	3.1:1 (2.8–3.3:1), SD 0.2:1	3.2:1 (2.9–3.8:1), SD 0.2:1	2.8:1 (2.5–3.4:1), SD 0.2:1	3.3:1 (2.6–4.5:1), SD 0.4:1
Opisthosoma length to body length ratio (%)	46 (43–48), SD 1	45 (41–49), SD 2	47 (43–56), SD 2	55 (50–61), SD 3
Aedeagus length	11 (9–13), SD 1	-	14 (12–20), SD 2	-
Vulva length	-	4 (3–5), SD 1	-	7 (5–10), SD 1

SD: standard deviation.

**Table 3:** Body (mean, range and SD, in μm) size for *Psorergatoides kerivoluae*.

Morphological Features	Present Study	Fain [17]	Giesen [4] *
	Females (*n* = 2)	Females (*n* = 6)	Females (*n* = 5)
Length of gnathosoma	24 (23–25), SD 1	No data	No data
Width of gnathosoma	34	No data	No data
Length of idiosoma	86 (83–88), SD 4	No data	No data
Width of idiosoma	100 (98–101), SD 2	(148–162) **	(148–162) **
Length of shield	81 (80–82), SD 1	No data	130
Width of shield	87 (84–89), SD 4	No data	126
Vulva length	12 (11–13), SD 1	No data	No data
Length of shield setae	less than 1	No data ***	less than 1
Length of gnathosomal setae	4	No data	3–4
Length of palpal tibial setae	13 (12–14), SD 1	15	(13–17)
Length of ventral setae	6 (5–6), SD 1	No data	(6–7)
Distance between ventral setae	16 (15–17), SD 1	No data	(16–18)
Total length of body	110 (106–113), SD 5	(170–186)	(170–186)

* It is probably that Giesen [4] obtained measurements of the specimens described by Fain [17]. ** Fain [17] and Giesen [4] measured the width of the body. *** Fain [17] described them as 5 pairs of very small circles each centered by a point which appears to be a very fine and very short hair.

**Table 4:** A checklist of skin mites in the Demodecidae and Psorergatidae families reported in bats.

Mites	Host Species (Family)	Habitat	Localities
Demodecidae			
*Demodex*			
*Demodex aelleni* Fain, 1960	*Myotis daubentonii* (Kuhl, 1918) (Vespertilionidae)	Patagium	Switzerland [18]
*Demodex artibei* Vargas, Bassols, Desch, Quintero et Polaco, 1995	*Artibeus aztecus* K. Andersen, 1906 (Phyllostomidae)	Upper and lower eyelids	Mexico [19]
*Demodex bicaudatus* Kniest et Lukoschus, 1981	*Macroglossus minimus* (E. Geoffroy, 1810) (Pteropodidae)	Eyelids (Meibomian glands)	Australia [20]
*Demodex carolliae* Desch, Lebel, Nuttingand et Lukoschus, 1971	*Carollia perspicillata* (Linnaeus, 1758) (Phyllostomidae)	Muzzle	Republic of Suriname [21]
*Demodex chiropteralis* Hirst, 1921	*Plecotus auritus* (Linnaeus, 1758) (Vespertilionidae)	Skin of the head	Great Britain [22], Poland [23]
*Demodex desmodi* Desch, 1994	*Desmodus rotundus* (E. Geoffroy, 1810) (Phyllostomidae)	Eyelids (Meibomian glands)	Republic of Suriname [24]
*Demodex longissimus* Desch, Nutting et Lukoschus, 1972	*Carollia perspicillata* (Phyllostomidae)	Eyelids (Meibomian glands)	Republic of Suriname [25]
*Demodex macroglossi* Desch, 1981	*Macroglossus minimus* (Pteropodidae)	Follicles of the eyelids and in a large dermal cysts on the neck	Australia [26]
*Demodex melanopteri* Lukoschus, Jongman et Nutting, 1972	*Eptesicus brasiliensis melanopterus* (Jentink, 1904) (Vespertilionidae)	Eyelids (Meibomian glands)	Republic of Suriname [27]
*Demodex mexicanus* Vargas, Bassols, Desch, Quintero et Polaco, 1995	*Corynorhinus mexicanus* (=*Plecotus mexicanus*) G. M. Allen, 1916 (Vespertilionidae)	Muzzle (sebaceous glands)	Mexico [19]
*Demodex molossi* Desch, Nutting et Lukoschus, 1972	*Molossus molossus* (Pallas, 1766) (Molossidae)	Eyelids (Meibomian glands)	Republic of Suriname [25]
*Demodex mystacina* Desch, 1989	*Mystacina tuberculata* Gray, 1843 (Mystacinidae)	Eyelids (Meibomian glands)	New Zealand [8]
*Demodex neoopisthosomae* Desch, Lukoschus et Nadchatram, 1986	*Eonycteris spelaea* (Dobson, 1871) (Pteropodidae)	Eyelids (Meibomian glands)	Malaysia [7]
*Demodex novazelandica* Desch, 1989	*Mystacina tuberculata* (Mystacinidae)	Eyelids	New Zealand [8]
*Demodex nycticeii* Desch, 1996	*Nycticeius humeralis* (Rafinesque, 1818) (Vespertilionidae)	Hairy skin of the body	USA [28]
*Demodex phyllostomatis* Leydig, 1859	*Phyllostomus hastatus* (Pallas, 1767) (Phyllostomidae)	Abdomen	Republic of Suriname [29]
*Demodex plecoti* Izdebska, Rolbiecki, Mierzyński et Bidziński, 2019	*Plecotus auritus* (Vespertilionidae)	Ear pinnae, wing membranes, posterior limbs, anterior limbs, tail	Poland [6]
*Demodex pusillus* Izdebska, Cierocka, Rolbiecki et Ciechanowski	*Nyctalus noctula* (Schreber, 1774) (Vespertilionidae)	Hairy skin of the body	Poland [present study]
*Demodex spelaea* Desch, Lukoschus et Nadchatram, 1986	*Eonycteris spelaea* (Pteropodidae)	Eyelids (Meibomian glands)	Malaysia [7]
*Ophtalmodex*			
*Ophtalmodex aritbei* Lukoschus et Nutting, 1979	*Artibeus lituratus* Olfers, 1818 (Phyllostomidae)	Corneal surface, eyelids fornixes	Republic of Suriname [30]
*Ophthalmodex australiensis* Woeltjes et Lukoschus, 1981	*Rhinonicteris aurantia* (Gray, 1845) (Rhinonycteridae)	Eyes	Australia [31]
*Ophthalmodex carolliae* Lukoschus, Woeltjes, Desch et Nutting, 1980	*Carollia perspicillata* (Phyllostomidae)	Ocular conjunctiva and the corneal beneath the eyelids	Republic of Suriname [32]
*Ophthalmodex juniatae* Veal, Giesen et Whitaker, 1984	*Myotis lucifugus* (Le Conte, 1831) (Vespertilionidae)	Ocular cavities	USA [33]
*Ophthalmodex molossi* Lukoschus, Woeltjes, Desch et Nutting, 1980	*Molossus molossus* (Molossidae)	Conjunctiva and the cornea beneath the eyelids	Republic of Suriname [32]
*Ophthalmodex wilsoni* Woeltjes et Lukoschus, 1981	*Vespadelus pumilus* (Gray, 1841) (Vespertilionidae)	Eyes	Australia [31]
*Pterodex*			
*Pterodex carolliae* Lukoschus, Woeltjes, Desch et Nutting, 1980	*Carollia perspicillata* (Phyllostomidae)	Area of the elbow	Republic of Suriname [34]
*Stomatodex*			
*Stomatodex corneti corneti* Fain, 1960	*Barbastella barbastellus* (Schreber, 1774) (Vespertilionidae)	Buccal mucosa	Belgium [18], Great Britain [35]
	*Nycteris* sp. (Nycteridae)	Buccal mucosa	Rwanda [18]
*Stomatodex corneti myotis* Fain, 1960	*Myotis dasycneme* (Boie, 1825) (Vespertilionidae)	In the oral mucosa, at the level of the soft palate, the lower surface of the tongue and the cheeks	Belgium [18]
	*Myotis myotis* (Borkhausen, 1797) (Vespertilionidae)	In the oral mucosa, at the level of the soft palate, the lower surface of the tongue and the cheeks	Belgium [18]
*Stomatodex rousetti* Fain, 1960	*Rousettus aegyptiacus* (Geoffroy, 1810) (Pteropodidae)	Buccal mucosa	Democratic Republic of Congo [18]
Psorergatidae			
*Psorergatoides*			
*Psorergatoides artibei* Lukoschus, Rosmalen et Fain, 1973	*Artibeus lituratus* (Phyllostomidae)	Epidermis of outside of ears	Republic of Suriname [36]
*Psorergatoides australiensis* Giesen, Lukoschus et Fain, 1982	*Vespadelus pumilus* (=*Eptesicus pumilus*) (Vespertilionidae)	Dactylopatagium between digits 3-4 on the dorsal side	Australia [37]
	*Vespadelus douglasorum* (*=Eptesicus douglasi*) Kitchener, 1976 (Vespertilionidae)	No data	Australia [37]
	*Nyctophilus arnhemensis* Johnson, 1959 (Vespertilionidae)	No data	Australia [37]
	*Nyctophilus walkeri* Thomas, 1892 (Vespertilionidae)	No data	Australia [37]
*Psorergatoides desmodus* Lukoschus, Louppen et Fauran, 1979	*Desmodus rotundus* (Phyllostomidae)	Wing membrane	French Guiana [38]
*Psorergatoides emballonurae* Fain, 1959	*Mosia nigrescens* (*=Emballonura nigrescens*) Gray, 1843 (Emballonuridae)	Wing membrane	New Guinea [39]
*Psorergatoides glossophagae* Lukoschus, Rosmalen et Fain, 1973	*Glossophaga soricina* Pallas, 1766 (Phyllostomidae)	Wing membrane	Republic of Suriname [36]
*Psorergatoides guyanensis* Lukoschus, Louppen et Fauran, 1979	*Rhinophylla pumilio* Peters, 1865 (Phyllostomidae)	Wing membrane	French Guiana [38]
*Psorergatoides hipposideros* Fain, 1959	*Hipposideros abae* J.A. Allen, 1917 (Hipposideridae)	Wing membrane	Democratic Republic of the Congo [39]
	*Hipposideros caffer* (Sundevall, 1846) (Hipposideridae)	Wing membrane	Democratic Republic of the Congo [39]
*Psorergatoides indicicola* Lukoschus, Rosmalen et Fain, 1973	*Saccopteryx canescens* Thomas, 1901 (Emballonuridae)	Epidermis around the end of second digit	Republic of Suriname [36]
	*Saccopteryx bilineata* Temminck, 1838 (Emballonuridae)	The end of the second digit	Republic of Suriname [36]
*Psorergatoides kerivoluae* Fain, 1959	*Kerivoula cuprosa* Thomas, 1912 (Vespertilionidae)	No data	Democratic Republic of the Congo [17]
	*Kerivoula lanosa* (*= Kerivoula harrisoni bellula*) A. Smith, 1847 (Vespertilionidae)	No data	Democratic Republic of the Congo [17]
	*Myotis muricola* (Gray, 1846) (Vespertilionidae)	Wing membrane	Borneo [39]
	*Myotis bocagii* Peters, 1870 (Vespertilionidae)	Wing membrane	Republic of Côte d’Ivoire [39]
	*Plecotus auritus* (Vespertilionidae)	Wing membrane, forehead region, ear canal	Belgium [39], Poland [40], present study
	*Myotis myotis* (Vespertilionidae)	Wing membrane	Poland [40]
	*Myotis mystacinus* (Kuhl, 1817) (Vespertilionidae)	No data	Malaysia [4] *
	*Myotis macropus* (Gould, 1854) (Vespertilionidae)	Wing membrane	Australia [41]
*Psorergatoides laviae* Fain, 1959	*Lavia frons* (É. Geoffroy, 1810) (Megadermatidae)	Wing membrane	Rwanda [17]
*Psorergatoides lonchorhina* Fain, 1959	*Lonchorhina aurita* Tomes, 1863 (Phyllostomidae)	Wing membrane	Venezuela [39]
	*Saccopteryx canescens* (Emballonuridae)	Ears	Venezuela [39]
*Psorergatoides molossi* Lukoschus, Rosmalen et Fain, 1973	*Molossus molossus* (Molossidae)	Epidermis of inner and outside of ears, on dorsal surface of wing membrane and tail membrane, on feet and tail	Republic of Suriname [36]
	*Molossus rufus* (*=Molossus ater*) É. Geoffroy Saint-Hilaire, 1805 (Molossidae)	No data	Republic of Suriname [36]
*Psorergatoides nyctali* Baker, 2005	*Nyctalus noctula* (Vespertilionidae)	Wing membrane	Great Britain [42], Poland [43]
*Psorergatoides nycteris* Fain, 1959	*Nycteris macrotis* Dobson, 1876 (Nycteridae)	Ears	Democratic Republic of the Congo [17]
	*Nycteris* sp. (Nycteridae)	Wing membrane, ears	Democratic Republic of the Congo [17]
*Psorergatoides peropteryx* Lukoschus, Louppen et Fauran, 1979	*Peropteryx macrotis* Wagner, 1843 (Emballonuridae)	Wing membrane	French Guiana [38]
	*Cormura brevirostris* (Wagner, 1843) (Emballonuridae)	Wing membrane	French Guiana [38]
*Psorergatoides rhinolophi* Fain, 1959	*Rhinolophus clivosus* Cretzschmar, 1828 (Rhinolophidae)	Wing membrane, nasal membrane, auricle	Democratic Republic of the Congo [17,39]
	*Rhinolophus hildebrandtii,* Peters 1878 (Rhinolophidae)	No data	Republic of Suriname [17]
	*Rhinolophus fumigatus* (*=Rhinolophus aethiops*) Rüppell, 1842 (Rhinolophidae)	No data	Angola [17]
	*Rhinolophus ferrumequinum* (Schreber, 1774) (Rhinolophidae)	No data	Belgium, France [39]
	*Rhinolophus hipposideros* (Bechstein, 1800) (Rhinolophidae)	No data	Belgium [39]
	*Rhinolophus affinis* Horsfield, 1823 (Rhinolophidae)	No data	Myanmar [39]
	*Rhinolophus euryale* (Blasius, 1853) (Rhinolophidae)	Wing membrane	Spain [44]
	*Rhinolophus mehelyi* Matschie, 1901 (Rhinolophidae)	No data	Italy [4]
*Psorergatoides surinamensis* Lukoschus, Louppen et Fauran, 1979	*Lophostoma brasiliense* (*=Tonatia nicarague*) Peters, 1866 (Phyllostomidae)	Wing membrane	Republic of Suriname [38]
	*Lophostoma carrikeri* (*=Tonatia carrikeri)* J.A. Allen, 1910 (Phyllostomidae)	No data	No data [4]
*Psorergatoides tadaridae* Giesen, Lukoschus et Nadchatram, 1982	*Mops mops* (Blainville, 1840) (Molossidae)	Dactylopatagium between digits 2-3 on the dorsal side	Malaysia [45]

* Questionable record/host; there is no *Myotis mystacinus* in Malaysia.

## References

[1] Boczek J., Błaszak C. (2005). Roztocze (Acari): Znaczenie w Życiu i Gospodarce Człowieka.

[2] Izdebska J.N., Krawczyk M., Buczek A., Błaszak C. (2012). Skin mites of mammals—The occurrence, significance and research prospects in Poland. Arthropods: The Medical and Economic Importance.

[3] Bochkov A.V. (2008). New observations on phylogeny of cheyletoid mites (Acari: Prostigmata: Cheyletoidea). Proc. Zool. Inst. RAS.

[4] Giesen K.M.T. (1990). A review of the parasitic mites of the family Psorergatidae (Cheyletoidea: Prostigmata: Acari) with hypotheses on the phylogenetic relationships of the species and species groups. Zool. Verh..

[5] Izdebska J.N., Rolbiecki L. (2020). The biodiversity of demodecid mites (Acariformes: Prostigmata), specific parasites of mammals with a global checklist and a new finding for *Demodex sciurinus*. Diversity.

[6] Izdebska J.N., Rolbiecki L., Mierzyński Ł., Bidziński K. (2019). Morphological and ontogenetic characteristics of *Demodex plecoti* sp. nov. (Acariformes: Demodecidae) from the brown long-eared bat *Plecotus auritus* (Chiroptera: Vespertilionidae), with comments on parasitism. Syst. Appl. Acarol..

[7] Desch C.E., Lukoschus F.S., Nadchatram M. (1986). Two new species of *Demodex* (Acari: Demodicidae) from the Meibomian glands of the tropical old world bat, *Eonycteris spelaea* (Chiroptera). Int. J. Acarol..

[8] Desch C.E. (1989). Two new species of *Demodex* (Acari: Demodicidae) from the New Zealand short-tailed bat, *Mystacina tuberculata* Gray, 1843 (Chiroptera: Mystacinidae). N. Z. J. Zool..

[9] Izdebska J.N., Rolbiecki L. (2014). New species of *Demodex* (Acari: Demodecidae) with data on parasitism and occurrence of other demodecids of *Rattus norvegicus* (Rodentia, Muridae). Ann. Entomol. Soc. Am..

[10] Izdebska J.N. (2004). *Demodex* spp. (Acari: Demodecidae) in brown rat (Rodentia: Muridae) in Poland. Wiad. Parazytol..

[11] Zhang Z.-Q. (2018). Repositories for mite and tick specimens: Acronyms and their nomenclature. Syst. Appl. Acarol..

[12] Nutting W.B. (1976). Hair follicle mites (*Demodex* spp.) of medical and veterinary concern. Cornell Vet..

[13] Izdebska J.N., Rolbiecki L. (2016). A new genus and species of demodecid mites from the tongue of a house mouse *Mus musculus*: Description of adult and immature stages with data on parasitism. Med. Vet. Entomol..

[14] Bush A.O., Lafferty K.D., Lotz J.M., Shostak A.W. (1997). Parasitology meets ecology on its own terms: Margolis et al. revisited. J. Parasitol..

[15] Wilson D.E., Reeder D.M. (2005). Mammals Species of the World: A Taxonomic and Geographic Reference.

[16] Taxonomic Information System (ITIS). http://www.itis.gov.

[17] Fain A. (1959). Les acariens psoriques parasites des Chauves-souris III: Le genre *Psorergates* Tyrell (Trombidiformes—Psorergatidae). Bull. Ann. Soc. R. Belg. Entomol..

[18] Fain A. (1960). Les acariens psoriques parasites des chauves-souris. XIII: La famille Demodicidae Nicolet. Acarologia.

[19] Vargas M., Bassols I.B., Desch C.E., Quintero M.T., Polaco O.J. (1995). Description of two new species of the genus *Demodex* Owen, 1843 (Acari: Demodecidae) associated with mexican bats. Int. J. Acarol..

[20] Kniest F.M., Lukoschus F.S. (1981). Parasites of Western Australia XIII: *Demodex bicaudatus* new species of demodicid mite from the Meibomian glands of the bat *Macroglossus minumus*. Rec. West. Aust. Mus..

[21] Desch C., Lebel R.R., Nutting W.B., Lukoschus F. (1971). Parasitic mites of Surinam: I. *Demodex carolliae* sp. nov. (Acari: Demodicidae) from the fruit bat *Carollia perspicillata*. Parasitology.

[22] Hirst S. (1921). On some new or little-known Acari, mostly parasitic in habit. Proc. Zool. Soc. Lond..

[23] Izdebska J.N., Rolbiecki L., Mierzyński Ł., Bidziński K. (2018). Demodecid mites (Acariformes, Demodecidae) in brown long-eared bat *Plecotus auritus* (Chiroptera, Vespertilionidae)—Second record in the world and systematic status of *Demodex chiropteralis* Hirst, 1921. Ann. Parasitol..

[24] Desch C. (1994). A new species of *Demodex* Owen, 1843 (Acari: Demodecidae) from the meibomian glands of the vampire bat *Desmodus rotundus* (E. Geoffroy, 1810) (Chiroptera: Phyllostomidae: Desmodontinae) from Surinam. Int. J. Acarol..

[25] Desch C., Nutting W.B., Lukoschus F.S. (1972). Parasitic mites of Surinam VII: *Demodex longissimus* n. sp. from *Carollia perspicillata* and *D. molossi* n. sp. from *Molossus molossus* (Demodicidae: Trombidiformes); meibomian complex inhabitants of neotropical bats (Chiroptera). Acarologia.

[26] Desch C.E. (1981). A new species of demodicid mite (Acari: Prostigmata) from Western Australia parasitic on *Macroglossus minimus* (Chiroptera: Pteropodidae). Rec. West. Aust. Mus..

[27] Lukoschus F.S., Jongman R.H.G., Nutting W.B. (1972). Parasitic mites of Surinam XII: *Demodex melanopteri* sp. n. (Demodicidae: Trombidiformes) from the Meibomian glands of the neotropical bat *Eptesicus melanopterus*. Acarologia.

[28] Desch C.E. (1996). *Demodex biciceii*: A new species of hair follicle mite (Acari: Demodecidae) from the evening bat, *Nycticeius humeralis* (Chiroptera: Vespertilionidae). Int. J. Acarol..

[29] Leydig F. (1859). Ueber Haarsackmilben und Krätzmilben. Arch. Naturgesch..

[30] Lukoschus F.S., Nutting W.B. (1979). Parasitic mites of Surinam XIII: *Ophthalmodex artibei* gen. nov., spec. nov. (Prostigmata: Demodicidae) from *Artibeus lituratus* with notes on pathogenesis. Int. J. Acarol..

[31] Woeltjes A.G.W., Lukoschus F.S. (1981). Parasites of Western Australia XIV two new species of *Ophtalmodex* Lukoschus and Nutting (Acarina: Prostigmata: Demodecidae) from the eyes of bats. Rec. West. Aust. Mus..

[32] Lukoschus F.S., Woeltjes A.G., Desch C.E., Nutting W.B. (1980). Parasitic mites of Surinam XXXV: Two new *Ophthalmodex* spp. (*O. carolliae*, *O. molossi*: Demodicidae) from the bats *Carollia perspicillata* and *Molossus molossus*. Int. J. Acarol..

[33] Veal R.A., Giesen K.M.T., Whitaker J.O. (1984). A new species of the genus *Ophtalmodex* Lukoschus & Nutting 1979 (Prostigmata: Demodicidae) from *Myotis lucifugus* (Chiroptera: Vespertilionidae). Acarologia.

[34] Lukoschus F.S., Woeltjes A.G.W., Desch C.E., Nutting W.B. (1980). Parasitic mites of Surinam XX: *Pterodex carolliae* gen. nov., spec. nov. (Demodicidae) from the fruit bat, *Carollia perspicillata*. Int. J. Acarol..

[35] Baker A.S., Craven J.C. (2003). Checklist of the mites (Arachnida: Acari) associated with bats (Mammalia: Chiroptera) in the British Isles. Syst. Appl. Acarol..

[36] Lukoschus F.S., Rosmalen P.G., Fain A. (1973). Parasitic mites of Surinam XI. Four new species of the genus *Psorergatoides* Fain, 1959, (Psorergatidae: Trombidiformes). Tijdschr. Entomol..

[37] Giesen K.M.T., Lukoschus F.S., Fain A. (1982). Parasites of Western Australia XV. A new species of *Psorergatoides* (Acarina: Psorergatidae) from Australian Bats. Rec. West. Aust. Mus..

[38] Lukoschus F.S., Louppen J.M.W., Fauran P. (1979). Parasitic mites of Surinam: XIV—New observations on the genus *Psorergatoides* Fain, 1959 (Psorergatidae: Trombidiformes), with a key to the known species. Int. J. Acarol..

[39] Fain A. (1959). Les acariens psoriques parasites des Chauves-souris IX: Nouvelles observations sur le genre *Psorergates* Tyrell. Bull. Ann. Soc. R. Belg. Entomol..

[40] Haitlinger R. (1979). External parasites of the Lower Silesian bats: V—Trombidiformes, Sarcoptiformes (Acarina). Wiad. Parazytol..

[41] Nelson L.J., Seeman O.D., Shinwari M.W. (2017). *Psorergatoides* cf. *kerivoulae* (Acari: Psorergatidae) induces cutaneous lesions on the wings of *Myotis macropus* (Chiroptera: Vespertilionidae). Syst. Appl. Acarol..

[42] Baker A.S. (2005). *Psorergatoides nyctali* (Prostigmata: Psorergatidae), a new mite species parasitizing the bat *Nyctalus noctula* (Mammalia: Chiroptera) in the British Isles. Syst. Appl. Acarol..

[43] Izdebska J.N., Fryderyk S., Ciechanowski M., Buczek A., Błaszak C. (2009). Spinturnix acuminatus (C.L. Koch, 1836), against the parasitofauna of the noctule bat *Nyctalus noctula* (Schreber, 1774). Arthropods: Invasions and Their Control.

[44] Lukoschus F.S. (1967). Krätzmilben an spanischen Kleinsaugern (Psorergatidae: Trombidiformes). Rev. Iber. Parasitol..

[45] Giesen K.M.T., Lukoschus F.S., Nadchatram M. (1982). Three new itch mites of the family Psorergatidae (Acari, Prostigmata) from Malaysian small mammals. Malay. Nat. J..

[46] Izdebska J.N., Rolbiecki L., Bielecki W. (2022). *Demodex bialoviensis* sp. nov. (Acariformes, Demodecidae) a new, specific parasite of the European bison *Bison bonasus* (Artiodactyla, Bovidae). Int. J. Parasitol. Parasites Wildl..

[47] Cierocka K., Izdebska J.N., Rolbiecki L. (2021). *Demodex crocidurae*, a new demodecid mite (Acariformes: Prostigmata) parasitizing the lesser white-toothed shrew and a redescription of *Demodex talpae* from european mole with data on parasitism in Soricomorpha. Animals.

[48] Izdebska J.N., Rolbiecki L., Fryderyk S. (2016). A new species of *Demodex* (Acari: Demodecidae) from the skin of the vibrissal area of the house mouse *Mus musculus* (Rodentia: Muridae), with data on parasitism. Syst. Appl. Acarol..

[49] Walter D.E., Lindquist E.E., Smith I.M., Cook D.R., Krantz G.W., Krantz G.W., Walter D.E. (2009). Order Trombidiformes. A Manual of Acarology.

[50] Wilson D.E., Mittermeier R.A. (2019). Handbook of the Mammals of the World.

[51] Ciechanowski M. (2020). Nietoperze—Chiroptera. Zoologia, Ssaki.

[52] Dietz C., von Helversen O., Nill D. (2009). Bats of Britain, Europe and Northwest Africa.

[53] Altringham J.D. (2011). Bats: From Evolution to Conservation.

